# The Negative Impact of Inflammation-Related Parameters in Prostate Cancer after Robot-Assisted Radical Prostatectomy: A Retrospective Multicenter Cohort Study in Japan (the MSUG94 Group)

**DOI:** 10.3390/jcm12247732

**Published:** 2023-12-17

**Authors:** Kazumasa Murase, Makoto Kawase, Shin Ebara, Tomoyuki Tatenuma, Takeshi Sasaki, Yoshinori Ikehata, Akinori Nakayama, Masahiro Toide, Tatsuaki Yoneda, Kazushige Sakaguchi, Jun Teishima, Kazuhide Makiyama, Takahiro Inoue, Hiroshi Kitamura, Kazutaka Saito, Fumitaka Koga, Shinji Urakami, Takuya Koie

**Affiliations:** 1 Department of Urology, Gifu University Graduate School of Medicine, Gifu 501-1194, Japan; murase.kazumasa.c1@f.gifu-u.ac.jp (K.M.); kawase.makoto.g5@f.gifu-u.ac.jp (M.K.); 2 Department of Urology, Hiroshima City Hiroshima Citizens Hospital, Hiroshima 730-8518, Japan; shinbone0127@yahoo.co.jp; 3 Department of Urology, Yokohama City University, Yokohama 236-0004, Japan; tatenuma@yokohama-cu.ac.jp (T.T.); makiya@yokohama-cu.ac.jp (K.M.); 4 Department of Nephro-Urologic Surgery and Andrology, Mie University Graduate School of Medicine, Tsu 514-8507, Japan; t-sasaki@clin.medic.mie-u.ac.jp (T.S.); tinoue28@clin.medic.mie-u.ac.jp (T.I.); 5 Department of Urology, University of Toyama, Toyama 930-0194, Japan; ikehata.y0226@gmail.com (Y.I.); hkitamur@med.u-toyama.ac.jp (H.K.); 6 Department of Urology, Dokkyo Medical University Saitama Medical Center, Koshigaya 343-8555, Japan; akinori@dokkyomed.ac.jp (A.N.); kzsaito@dokkyomed.ac.jp (K.S.); 7 Department of Urology, Tokyo Metropolitan Cancer and Infectious Diseases Center Komagome Hospital, Tokyo 113-8677, Japan; m.toide@gmail.com (M.T.); f-koga@cick.jp (F.K.); 8 Department of Urology, Seirei Hamamatsu General Hospital, Hamamatsu 430-8558, Japan; yonet@sis.seirei.or.jp; 9 Department of Urology, Toranomon Hospital, Tokyo 105-8470, Japan; kazhimaro@gmail.com (K.S.); shinji.urakami@toranomon.gr.jp (S.U.); 10 Department of Surgery, Division of Urology, Kobe University Graduate School of Medicine, Kobe 650-0017, Japan; teishimaj@yahoo.co.jp

**Keywords:** multicenter cohort study, prostate cancer, robot-assisted radical prostatectomy, inflammatory-related score, oncological outcomes

## Abstract

*Background and Objectives*: We aimed to examine the relationship between the inflammation-related parameters, such as the neutrophil-to-lymphocyte ratio (NLR), and the pathological findings and biochemical recurrence (BCR) in patients with prostate cancer (PCa) undergoing robot-assisted radical prostatectomy (RARP). *Materials and Methods*: A retrospective multicenter cohort study of patients with PCa who underwent RARP at 10 institutes in Japan was conducted. This study enrolled 3195 patients. We focused on patients undergoing RARP who underwent the preoperative measurement of their inflammation-related parameters and who did not receive any neo- or adjuvant therapy. Data on the pre- and postoperative variables for the enrolled patients were obtained. The primary endpoint of this study was the association between BCR and the inflammation-related parameters after RARP. The secondary endpoint was the association between the inflammation-related parameters and the pathological diagnosis of PCa. *Results*: Data from 2429 patients with PCa who met the study’s eligibility criteria were analyzed. The median follow-up period was 25.1 months. The inflammation-related parameters were divided into two groups, and cutoff values were determined based on the receiver operating characteristics. There were no statistically significant differences in biochemical recurrence-free survival for any of the parameters. In the univariate analysis, the NLR was predictive of pathological T3 and lymphovascular invasion; however, there were no significant differences in the multivariate analysis. *Conclusions*: The inflammation-related parameters did not significantly affect the incidence of BCR, at least among patients with PCa who underwent RARP.

## 1. Introduction

The World Health Organization (WHO) reported that prostate cancer (PCa) is a common solid tumor in men; 1,414,259 new patients were diagnosed with PCa; and 375,304 deaths were PCa-related worldwide in 2020 [1]. Therefore, various surrogate markers and nomograms have been investigated to predict cancer progression and prognosis [2,3,4,5].

The relationships between inflammation-related parameters and tumorigenesis have been widely studied in various tumors [6,7,8,9,10]. In particular, PCa, which is a systemic, multifactorial disease, has been extensively investigated [11,12,13,14,15,16]. Inflammatory cells and mediators in the tumor microenvironment are known to mediate the inflammatory response and further interact with the tumor and peritumoral cells in an autocrine and/or paracrine manner [17]. The tumor microenvironment, which consists mainly of immune cells surrounding the cancer cells, has been suggested as a potential prognostic biomarker for PCa [18]. Therefore, inflammation-related parameters may be considered to contribute to the diagnosis and treatment of PCa as well as to the prognosis of the disease [18]. Lymphocyte-related markers of inflammation, such as C-reactive protein (CRP), the neutrophil-to-lymphocyte ratio (NLR), the platelet-to-lymphocyte ratio (PLR), the lymphocyte-to-monocyte ratio (LMR), and the systemic immune inflammation index (SII), are commonly used as markers of systemic inflammatory responses [1]. However, there is still no unified understanding of how inflammation-related parameters are related to PCa progression and oncological outcomes.

Therefore, we aimed to examine the relationship between the inflammation-related parameters and the pathological findings and BCR in patients with PCa who underwent robot-assisted RP.

## 2. Materials and Methods

### 2.1. Patient Population

This research was authorized by the Institutional Review Board of Gifu University (Approval No.: 2021-A050). Due to the retrospective study design, the requirement for patient consent was waived. In accordance with the provisions of the Japanese Ethics Committee and Ethical Guidelines, in the case of retrospective and/or observational research, information from these studies is disclosed to the public through existing documents and other materials. Detailed information on this study is available only in Japanese at https://www.med.gifu-u.ac.jp/visitors/disclosure/docs/2021-B039.pdf (accessed on 6 October 2023).

A retrospective multicenter cohort study of patients who underwent robot-assisted radical prostatectomy (RARP) for PCa at 10 Japanese institutes from September 2012 to August 2021 was conducted. The following preoperative information was collected from the patients enrolled in the study: age, height, weight, Eastern Cooperative Oncology Group Performance Status (ECOG-PS) [19], preoperative serum prostate-specific antigen (PSA) level, prostate volume, biopsy Gleason grade group (GG) [20], clinical T stage, and National Comprehensive Cancer Network risk stratification [21]. All patients underwent tumor disease classification according to the American Joint Committee on Cancer’s 8th edition of the Cancer Staging Manual [22]. No data were obtained in this study on whether enrolled patients received evaluation with magnetic resonance imaging before prostate biopsy. For biopsy GG, specimens were assessed in accordance with the International Society of Urologic Pathology (ISUP) 2014 guidelines [20].

All enrolled patients underwent RARP in this study. Pelvic lymph-node dissection (PLND), extent of PLND, and nerve sparing were decided at the individual surgeon’s or institution’s discretion.

### 2.2. Pathological Analysis

Based on ISUP2014 guidelines, all prostatectomy specimens were sectioned in accordance with the whole-mount staining method and were assessed for PCa [20]. A pathologist vertically sectioned the prostate apex against the prostatic urethra. The pathologist cut a conical section of the bladder neck margin from the specimen and dissected it perpendicularly. For the remaining prostatic tissue, complete sections were made along a plane perpendicular to the urethral axis at 3–5 mm intervals.

### 2.3. Follow-Up Schedule

Patients enrolled in this study were monitored for serum PSA and testosterone levels at 3-month intervals postoperatively. BCR was diagnosed when the postoperative serum PSA level increased to 0.2 ng/mL. If the postoperative PSA level did not fall to 0.2 ng/mL at any time, the date of RP was defined as the date of BCR onset.

### 2.4. Endpoints and Statistics

The primary endpoint of this study was the association between BCR and inflammation-related parameters after RARP. The secondary endpoint was the association between inflammation-related parameters and the pathological diagnosis of PCa. Data were analyzed using JMP 14 software (SAS Institute Inc., Cary, NC, USA). We assessed continuous and categorical variables using the Wilcoxon rank-sum test and Fisher’s exact test. BRFS after RARP was evaluated using the Kaplan–Meier method, and the association between BCR and subgroup classification was analyzed based on the log-rank test. Multivariate analysis was carried out using a Cox proportional hazards model. Using receiver operating characteristic (ROC) curve analysis, the cutoff values of the clinical variables were decided [23]. All two-sided *p*-values were employed, and statistical significance was defined as a *p*-value < 0.05.

## 3. Results

### 3.1. Patient Characteristics

Table 1 lists the preoperative and postoperative covariates of the patients enrolled in this study. A total of 3195 patients were enrolled in this study. Of these, 571 (17.9%) patients who received neoadjuvant or adjuvant therapy and 195 (6.1%) patients with missing NLR data were excluded from the analysis. Finally, we analyzed the oncologic outcome and perioperative covariates for 2429 (76.0%) patients. Although several surgeons from each institution performed RARP, this study did not evaluate the differences in the number of cases per surgeon.

### 3.2. Oncological Outcomes

By the end of the follow-up period, BCR occurred in 266 patients (11.0%), radiological recurrence occurred in 22 (0.9%), and castration-resistant PCa occurred in 11 (0.5%); however, none of the patients died of PCa.

**Figure 1:**
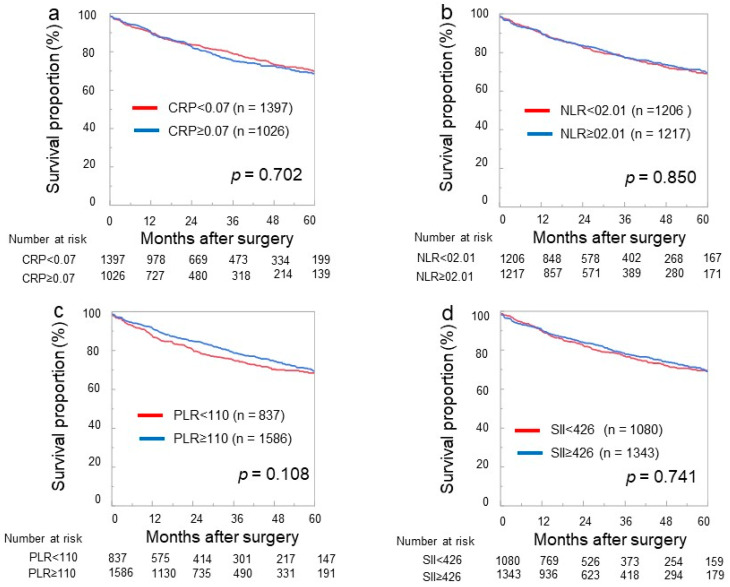
Kaplan–Meier estimates of biochemical recurrence-free survival when C-reactive protein (CRP) was stratified with a cutoff value of 0.07 mg/dL (**a**), neutrophil-to-lymphocyte ratio (NLR) was stratified with a cutoff value of 2.01 (**b**), platelet-to-lymphocyte ratio (PLR) was stratified with a cutoff value of 110 (**c**), and systemic immune inflammation index (SII) was stratified with a cutoff value of 426 (**d**). The 2-year BRFS rate was 91.5% for CRP < 0.07 mg/dL and 90.4% for CRP ≥ 0.07 mg/dL (*p* = 0.702 (**a**)), 89.7% for NLR < 2.01 and 91.9% for NLR ≥ 2.01 (*p* = 0.850 (**b**)), 89.2% for PLR < 110 and 92.5% for PLR ≥ 110 (*p* = 0.108 (**c**)), and 89.4% for SII < 426 and 92.2% for SII ≥ 426 (*p* = 0.741 (**d**)).

Figure 1 shows the relationship between BCR and the inflammation–related parameters, including CRP, NLR, PLR, and SII. The cutoff values of the inflammation-related parameters, using an ROC curve analysis, were 0.07 mg/dL for CRP, 2.01 for NLR, 110 for PLR, and 426 for SII. The patients were divided into two groups based on the cutoff values for each inflammation-related parameter, and the BRFS was examined for each parameter. The 2-year BRFS rate was 91.5% for CRP < 0.07 mg/dL and 90.4% for CRP ≥ 0.07 mg/dL (*p* = 0.702; Figure 1a), 89.7% for NLR < 2.01 and 91.9% for ≥2.01 (*p* = 0.850; Figure 1b), 89.2% for PLR < 110 and 92.5% for PLR ≥110 (*p* = 0.108; Figure 1c), and 89.4% for SII < 426 and 92.2% for SII ≥ 426 (*p* = 0.741; Figure 1d). There were no significant differences in the BRFS among the four inflammation-related parameters.

**Figure 2:**
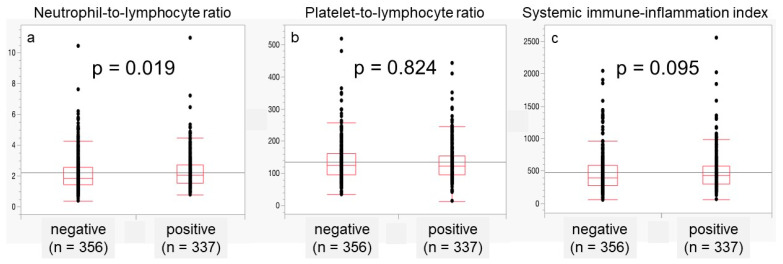
The association of NLR, PLR, and SII with lymphovascular invasion (LVI) in locally advanced PCa above pT3. The median NLR was 2.069 for patients with positive LVI and 1.850 for those with negative LVI (**a**). The median PLR values of LVI-positive and -negative patients were 123 and 125, respectively (**b**). The median SII was 426 for patients with positive LVI and 392 for those with negative LVI (**c**).

Subsequently, we examined the association of NLR, PLR, and SII with lymphovascular invasion (LVI) in patients with locally advanced PCa with ≥ pT3 (Figure 2). The median NLR was 2.069 in patients with positive LVI and 1.850 in those with negative LVI, which was significantly higher in patients with positive LVI (*p* = 0.019; Figure 2a). PLR and SII did not differ significantly between LVI-positive and LVI-negative patients.

Table 2 shows the patient demographics of the two groups of cases divided by a cutoff value of 2.01 NLR. Compared to the group with NLR ≥ 2.01, age, CRP, PLR, SII, and LVI were significantly lower in the group with NLR < 2.01. By contrast, BMI and BCR were significantly lower in patients with NLR ≥ 2.01 than in those with NLR < 2.01. In addition, radiographic recurrence and the rate of progression to CRPC tended to be lower in these patients.

A univariate analysis showed significant differences in the clinical T stage, biopsy GG, initial PSA, and NLR. In a multivariate analysis, the clinical T stage, biopsy GG, and initial PSA were significant predictors of locally advanced disease and LVI in patients with PCa undergoing RARP; however, the NLR was not significantly different in this study (Table 3).

## 4. Discussion

Several studies have shown that many types of malignant neoplasms play an important role in the carcinogenic process and survival with respect to systemic inflammation [6,7,8,9,10,11,12,13,14,15,16,24]. Tumor cells themselves release cytokines and chemokines, which are thought to circulate and induce a systemic inflammatory response [25], resulting in changes in neutrophils, lymphocytes, and platelets [26]. Neutrophils play distinct roles in regulating tumor cell proliferation and angiogenesis [27,28]. Lymphocytes play an important role in the regulation and integration of systemic immune responses [26]. Platelets have been shown to protect tumor cells from adhering to and being excluded from the vascular endothelium, thus contributing to their survival and spread [29,30]. In fact, inflammatory markers have been reported to be associated with tumor stage and grade, oncological outcome, and prognosis in various types of malignant neoplasms [5,6,7,8,9,10]. Because neutrophils, lymphocytes, and platelets have these functions with respect to tumor cells, the role of the inflammation-related parameters in PCa is of interest.

Inflammation-related parameters such as NLR, PLR, and SII have been focused on as potential useful markers for the assessment of systemic inflammation, and previous studies have suggested that these biomarkers may be one of the prognostic predictive parameters for PCa [12,26,31]. In a study of 291 patients with pathologically confirmed localized PCa who underwent RP, 114 (39.2%) developed BCR within a median follow-up of 48 months [12]. The incidence of BCR was significantly higher in the high NLR group (50.7%) and the high SII group (48.77%) than in the low NLR and SII groups [12]. A Kaplan–Meier analysis showed that patients with a high SII and NLR had a significantly poorer BRFS (*p* < 0.001 and *p* < 0.001, respectively) [12]. In the multivariate analysis, both a high SII ≥ 528.54 (HR, 4.251; 95%CI, 2.262–9.037; *p* < 0.001) and NLR ≥ 2.62 (HR, 4.787; 95%CI, 2.339–9.798; *p* < 0.001) were significant predictors of BCR after RP (*p* < 0.001 and *p* < 0.001, respectively) [12]. A high derived NLR (*p* = 0.044), high PLR (*p* = 0.028), and low prognostic nutrition index (PNI; *p* = 0.004) were associated with a poor BRFS in 380 patients who underwent RP for high-risk localized PCa without neoadjuvant androgen deprivation therapy [26]. In particular, the PNI is an independent predictive factor for BCR (HR, 0.56; 95%CI, 0.35–0.90; *p* = 0.016) [26]. The pooled results from 32 studies, including 21,949 patients, showed that higher pretreatment NLRs (HR, 1.55; 95%CI, 1.37–1.76), PLRs (HR, 1.72; 95%CI, 1.36–2.18), and neutrophil (HR, 1.10; 95%CI, 1.03–1.18) and monocyte counts (HR, 1.75; 95%CI, 1.36–2.25) were associated with poorer OS, while a higher pretreatment LMR correlated with better OS (HR, 2.27; 95%CI, 1.76–2.94) [30]. Furthermore, a higher NLR (HR, 1.62; 95%CI, 1.29–2.04) and monocyte count (HR, 1.75; 95%CI, 1.36–2.25) and a lower LMR (HR, 2.18; 95%CI, 1.58–3.02) predicted worse PFS [31]. These studies suggest that the pretreatment inflammation-related parameters may be useful for predicting oncological outcomes in patients with PCa after RP.

Conversely, several studies have reported no association between the inflammation-related parameters and oncological outcomes in patients with PCa after RP [13,15,16,32,33]. In a study of 668 patients with localized PCa who underwent RP, the NLR was not associated with the pathological status, including the GG (*p* = 0.159), lymph-node involvement (*p* = 0.15), or surgical margin status (*p* = 0.159) [13]. In addition, a high NLR was not statistically associated with MFS, CSS, OS, BRFS, or PCa grade [13]. In a study evaluating the NLR in 73 patients undergoing RP for PCa, there was no significant correlation between NLR and tumor grade, pathologic T stage, lymph-node metastasis, or surgical margin status [16]. Maeda et al. [16] found no significant correlation between NLR and tumor grade (*p* = 0.834), pathologic T stage (*p* = 0.082), lymph-node involvement (*p* = 0.062), or surgical margin status (*p* = 0.772). Based on an ROC curve analysis predicting biochemical recurrence after RP, a possible NLR cutoff point was determined to be 2.88 or 3.88; however, none of these cutoff points accurately predicted prognosis [16]. With respect to 237 patients with PCa who underwent RP, for the investigation of the relationship between pretreatment NLR and BCR, the NLR was not an independent predictor of BCR in a multivariate analysis, even though patients with a higher NLR had a significantly shorter BRFS than those with a lower NLR (*p* = 0.019) [32]. Additionally, the NLR was not statistically associated with upstaging, upgrading, or BCR (*p* = 0.368, *p* = 0.573, and *p* = 0.504, respectively) in a study of 217 patients with PCa [33]. In the present study, the NLR tended to be higher in patients with pathological T3 and LVI, although the multivariate analysis showed no significant difference. Therefore, further validation of the possibility that the NLR reflects the characteristics of PCa and the local immune microenvironment is needed because there are contradictory data on this issue.

This research has certain limitations. First, this study had a retrospective multicenter design and may have been susceptible to potential selection bias caused by diagnostic and surgical approach differences among the participating centers. Second, the relatively short follow-up period and varying intervals among each institution may have precluded the consideration of its true usefulness as a surrogate marker for BCR in analyzing the inflammation-related parameters. Finally, a prospective multicenter study is needed to evaluate the usefulness of the inflammation-related parameters in predicting the oncological outcomes of PCa.

## 5. Conclusions

The inflammation-related parameters, such as CRP, NLR, PLR, and SII, were not significantly associated with the incidence of BCR, at least among patients with PCa who underwent RARP. Further investigation into the inflammation-related parameters in RARP is warranted.

## Figures and Tables

**Table 1:** Pre- and postoperative covariates for enrolled patients.

Age (year, median, IQR)	68 (64–72)
Body mass index (kg/m^2^, median, IQR)	23.5 (21.7–25.5)
ECOG-PS (number, %)	
0	2356 (97.0)
1	73 (3.0)
Initial PSA (ng/mL, median, IQR)	7.6 (5.6–11.3)
Prostate volume (mL, median, IQR)	30 (23–40)
Biopsy Gleason grade group (number, %)	
1	529 (21.8)
2	770 (31.7)
3	511 (21.0)
4	468 (19.3)
5	151 (6.2)
Clinical T stage (number, %)	
1	494 (20.4)
2	1769 (72.9)
3	164 (6.8)
Unknown	2 (0.08)
CRP (mg/dL, median, IQR)	0.07 (0.03–0.11)
NLR (median, IQR)	2.01 (1.54–2.73)
PLR (median, IQR)	129 (101–166)
SII (median, IQR)	426 (306–587)
Pathological Gleason grade group (number, %)	
1	188 (7.8)
2	982 (40.6)
3	788 (32.6)
4	260 (10.7)
5	201 (8.3)
Unknown	10 (4.1)
Pathological T stage (number, %)	
0	3 (0.1)
2	1732 (71.3)
3	688 (28.3)
4	5 (0.2)
Unknown	1 (0.04)
Pelvic lymphadenectomy (number, %)	
Not performed	793 (32.6)
Performed	1636 (67.4)
Unknown	1 (0.04)
LVI (number, %)	740 (30.5)
Follow-up period (months, median, IQR)	25.1 (11.9–49.7)

IQR, interquartile range; ECOG-PS, Eastern Cooperative Oncology Group Performance Status; PSA, prostate-specific antigen; CRP, C-reactive protein; NLR, neutrophil-to-lymphocyte ratio; PLR, platelet-to-lymphocyte ratio; SII, systemic immune inflammation index; and LVI, lymphovascular invasion.

**Table 2:** Pre- and postoperative covariates for enrolled patients divided into two groups with a cutoff value of 2.01 for neutrophil-to-lymphocyte ratio.

	NLR < 2.01 (*n* = 1211)	NLR ≥ 2.01 (*n* = 1218)	*p*-Value
Age (year, median, IQR)	68 (64–72)	69 (65–73)	<0.001
Body mass index (kg/m^2^, median, IQR)	23.8 (22.0–25.8)	23.3 (21.5–25.3)	<0.001
ECOG-PS (number, %)			0.420
0	1178 (97.3)	1178 (96.7)	
1	33 (2.7)	40 (3.3)	
Initial PSA (ng/mL, median, IQR)	7.5 (5.5–11.2)	7.7 (5.7–11.3)	0.107
Prostate volume (mL, median, IQR)	30 (22–39)	30 (23–40)	0.201
Biopsy Gleason grade group (number, %)			0.245
1	251 (20.7)	278 (22.8)	
2	397 (32.8)	373 (30.6)	
3	240 (19.8)	271 (22.2)	
4	247 (20.4)	221 (18.1)	
5	76 (6.3)	75 (6.2)	
Clinical T stage (number, %)			0.327
1	232 (19.2)	262 (21.6)	
2	894 (73.8)	875 (72.0)	
3	85 (7.0)	79 (6.5)	
Unknown	0 (0)	2 (0.2)	
CRP (mg/dL, median, IQR)	0.06 (0.03–0.10)	0.07 (0.03–0.12)	0.001
PLR (median, IQR)	107 (86–132)	153 (125 –93)	< 0.001
SII (median, IQR)	310 (241–382)	582 (475–747)	< 0.001
Pathological Gleason grade group (number, %)			0.294
1	94 (7.8)	94(7.7)	
2	498 (41.3)	484 (39.9)	
3	369 (30.6)	419 (34.5)	
4	137 (11.4)	123 (10.1)	
5	107 (8.9)	94 (7.7)	
Unknown	6 (0.5)	4 (0.3)	
Pathological T stage (number, %)			0.375
0	1 (0.1)	2 (0.2)	
2	846 (69.9)	886 (72.7)	
3	360 (29.8)	328 (26.9)	
4	3 (0.3)	2 (0.2)	
Unknown	1 (0.1)	0	
Pelvic lymphadenectomy (number, %)			0.349
Not performed	386 (31.9)	407 (33.4)	
Performed	822 (67.9)	810 (66.5)	
Unknown	3 (0.2)	1 (0.1)	
LVI (number, %)	343 (28.3)	397 (32.6)	0.022
BCR (number, %)	148 (12.3)	118 (9.7)	0.037
Radiological recurrence (number, %)	14 (1.2)	8 (0.7)	0.199
CRPC (number, %)	7 (0.6)	4 (0.3)	0.365
Follow-up period (months, median, IQR)	26.0 (11.9–49.9)	24.2 (11.8–49.5)	0.220

NLR, neutrophil-to-lymphocyte ratio; IQR, interquartile range; ECOG-PS, Eastern Cooperative Oncology Group Performance Status; PSA, prostate-specific antigen; CRP, C-reactive protein; PLR, platelet-to-lymphocyte ratio; SII, systemic immune inflammation index; LVI, lymphovascular invasion; BCR, biochemical recurrence; and CRPC, castration-resistant prostate cancer.

**Table 3:** Univariate and multivariate analyses for predicting prostate cancer with ≥ pathological T3 and lymphovascular invasion.

Variables	Univariate		Multivariate	
	OR (95%CI)	*p*-Value	OR (95%CI)	*p*-Value
Clinical T stage (continuous)	2.644 (2.065–3.386)	< 0.001	1.745 (1.350–2.256)	<0.001
Biopsy Gleason grade group (continuous)	1.655 (1.501–1.826)	< 0.001	1.498 (1.351–1.660)	<0.001
Initial PSA (continuous)	1.062 (1.048–1.077)	< 0.001	1.049 (1.034–1.064)	<0.001
NLR (continuous)	1.224 (1.029–1.455)	0.022	1.204 (0.945–1.533)	0.133

OR, odds ratio; CI, confidence interval; PSA, prostate-specific antigen; and NLR, neutrophil-to-lymphocyte ratio.

## Data Availability

The data presented in this study are available on request from the corresponding author. The data are not publicly available due to privacy and ethical reasons.
